# Molecular portrait of squamous cell carcinoma of the bovine horn evaluated by high-throughput targeted exome sequencing: a preliminary report

**DOI:** 10.1186/s12917-020-02683-y

**Published:** 2020-11-26

**Authors:** Dhruv Bhatia, Ankit Hinsu, Ketankumar Panchal, Pritesh Sabara, Subhash Jakhesara, Prakash Koringa

**Affiliations:** Department of Animal Biotechnology, College of Veterinary Sciences & A.H., Anand Agricultural University, Anand, 388001 India

**Keywords:** Horn cancer, Cancer, SCC, Squamous cell carcinoma, Exome sequencing, KRT8

## Abstract

**Background:**

Squamous Cell Carcinoma of horn, also known as horn cancer, is a prevailing type of cancer in cattles especially *Bos indicus*. It is one of the most prevalent disease in Indian bullocks often resulting in death and huge economic losses to farmers. Here, we have reported the use of targeted exome sequencing to identify variants present in horn cancer affected horn mucosa tissue and blood of the same animal to identify some of the prevalent markers of horn cancer.

**Results:**

We have observed higher number of variants present in tissue as compared to blood as well as among cancer samples compared to samples from normal animals. Eighty six and 1437 cancer-specific variants were identified among the predicted variants in blood and tissue samples, respectively. Total 25 missense variants were observed distributed over 18 genes. *KRT8* gene coding for Keratin8, one of the key constituents of horn, displayed 5 missense variants. Additionally, three other genes involved in apoptosis pathway and two genes involved in antigen presentation and processing also contained variants.

**Conclusions:**

Several genes involved in various apoptotic pathways were found to contain non-synonymous mutations. Keratin8 coding for Keratin, a chief constituent of horn was observed to have the highest number of mutations. In all, we present a preliminary report of mutations observed in horn cancer.

**Supplementary Information:**

The online version contains supplementary material available at 10.1186/s12917-020-02683-y.

## Background

Cancer is a complex disease caused by multiple etiological factors and affects almost every species of mammals present on earth. Various types of cancer prevalent in farm animals causes moderate to severe economic losses to the farmers. Squamous Cell Carcinoma (SCC) is one of the most common cancer capable of metastatic spread and is observed in various forms across many animals and humans [1–3]. The accumulation of genetic and epigenetic alterations in cancer cells endows them with unwanted proliferative and metastatic potential.

Horn cancer is a widespread cancer reported in Indian zebu cattle (*Bos indicus*) with higher frequency in Kankrej breed than other zebu cattle, nondescript cattle or crossbred [4]. It is a type of SCC with poorly defined genetic landscape, which arise from pseudo stratified columnar epithelium of the horn core mucosa, reported only in *Bos indicus*. Horn Cancer often results in death of an animal in the event of metastasis [5]. In India, horn cancer affects approximately 1% of the cattle population and accounts for 83.34% of total tumours reported [6]. A few cases of horn cancer were also reported from Sumatra, Brazil, and Iraq. Castrated male animals i.e. bullocks make up 95% of the affected animals and cows 5%, and rarely observed in bulls, buffaloes, sheep and goats [7, 8].

An era of Next Generation Sequencing (NGS) began in the last decade providing an opportunity for simultaneous sequencing of millions of DNA fragments without previous sequence knowledge. This advancement in technology has been a true revolution compared with the traditional sequencing methods. Particularly, Whole Exome Sequencing is a powerful method designed to rapidly investigate all the coding sequences in genome at a base resolution, permitting to reveal a wide spectrum of genetic variations, especially SNP [9].

Here, we employed high throughput targeted exome sequencing using Illumina MiSeq for identification of mutations associated with squamous cell carcinoma of horn in Kankrej (*Bos indicus*) bullock. Previous studies on horn cancer have identified various SNPs and differential expression of genes using RNAseq or transcriptome sequencing [5, 7, 8, 10–14]. As per our knowledge, this is the first study of exome sequencing in bovine horn cancer (as well as other types of cancers in bovines).

## Methods

### Sample collection

Twenty-five Kankrej breed bullocks which were clinically diagnosed with horn cancer were considered for this study. Additionally, 5 Kankrej bullocks without horn cancer but with horn fracture were included as normal-horn sample totaling 30 animals were considered for this study. Notably, no animals were recruited or purchased for the study. All the animals had to undergo a corrective/curative surgery which involves amputation of their horn. The owners of the animals were informed about the experiment and the samples were collected from the amputated horn during the corrective/curative amputation surgery at the veterinary clinics across the Gujarat state of India. No animals were euthanised or died during the surgery. As such cases are very few, a greater number of samples could not be included in the current study.

Horn core mucosa was collected in RNAlater (Qiagen, Germany) during the surgery. The samples were stored in liquid nitrogen and transferred to laboratory. Additionally, blood samples were also collected in sterile EDTA vacutainer and transferred to laboratory in refrigerated condition.

### DNA extraction

DNA was extracted from blood and tissue samples using Qiagen DNAeasy Blood & Tissue kit (Qiagen, Germany) following manufacturer’s protocol. DNA quantity was checked on Qubit 3.0 (ThermoFisher Scientific, USA) and DNA quality was assessed by agarose gel electrophoresis.

### Library preparation and sequencing

Illumina compatible library was prepared using Illumina TruSeq Nano DNA LT library prep kit (Illumina, USA) following manufacturer’s protocol for 550 bp insert fragment size chemistry. Libraries were checked on Agilent 2100 Bioanalyzer (Agilent, USA) and quantified using Qubit 3.0.

Custom probes were designed and synthesized by Roche diagnostics (Roche, Switzerland) based on cattle genome bosTau7 assembly and annotation from UCSC Genome browser’s RefGene. Overall, 125,679 exons, 16,574 5’UTRs and 14,084 3’UTRs were targeted. Overall, 30,916,291 bases (30.9 Mb) were specifically included in design. The probe design and details are mentioned in our previous studies [15, 16]. The probes were used to enrich targets by using Roche NimbleGen SeqCap EZ Libraries capture as per manufacturer’s protocol. Briefly, probes were hybridised with libraries, captured using beads, amplified captured DNA using LM-PCR and purification of amplified captured library. Final captured-amplified library was checked on Agilent 2100 Bioanalyzer and quantified using Qubit 3.0. Libraries were diluted, normalised, and sequenced on Illumina MiSeq using 2 × 250 v2 chemistry.

### Data analysis

All the raw data was visualised using FastQC [17] and filtered/trimmed using Prinseq-lite [18] perl script. Data was trimmed 12 bases from 3′ end and sequences with mean quality score less than 30 were discarded. High quality data was mapped to the genome of *Bos taurus* assembly Btau_4.6.1/bosTau7 using bwa mem v0.7.5a [19]. Samtools suite v0.1.19 [20] was used to remove multiple mapped reads, sort and convert .sam files to .bam files. Cleaned bam files were used for variant calling using Freebayes v0.9.20 [21] (minimum read depth 10, minimum mapping quality 30, minimum base quality 20). Cancer-specific variants were identified by filtering predicted variants on the criteria: should be present in at least 80% of Cancer samples and the variant should be absent in at least 60% of sample. Further, frequency of reference and alternate nucleotide for all cancer-specific variant in horn normal samples were checked to verify. Based on nucleotide frequency, variants were further filtered if a greater number of horn normal samples showed alternate nucleotide although having mapped reads less than 10 at that position. The effect of SNPs was checked using SNPEff v4.3a [22] trained against BTau4.6.1 assembly and RefSeq gene annotation available at UCSC genome browser. The proteins were scanned using ScanProsite, which compares with PROSITE, for presence and position of domains [23]. PROVEAN web server was used to check the effect of these variants on the structure of proteins [24].

## Results

The study was aimed at identification of SNPs and somatic mutations associated with SCC of horn in bullocks. Illumina sequencing generated total 148 GB data with an average of 4.92 million paired-reads per sample (Table S1). All the reads were mapped to bosTau7 genome with bwa. On an average 99% paired-reads per sample mapped to the genome with an average 45.22% mapping to the targeted regions (Table S1). Further, variants were identified using Freebayes present within the designed regions. Number of variants per sample ranged from 15,136 to 69,071 in blood samples and 13,106 to 67,073 in tissue samples. An average of 35,768; 45,845; 45,089; and 25,414 variants were identified in cancer-blood, cancer-tissue, normal-blood and normal-tissue groups, respectively (Table S1).

Cancer-specific variants were filtered based on the criteria: present in at least 80% of cancer samples and at the most 40% of normal samples has the same variant. This resulted in 86 cancer-specific variants in blood samples and 1436 cancer-specific variants in tissue samples. Further, a manual curation and verification of these variants was performed including positions with nucleotide frequency with read depth less than 10. This resulted in final 30 and 96 cancer-specific variants in blood and tissue samples, respectively. Annotation with SNPEff revealed 7 and 28 synonymous variants & 4 and 21 non-synonymous (missense) variants present in exonic region from blood and tissue samples, respectively (Table 1). Other variants were either intergenic or present in intron or present in UTR region. All the intergenic variants were located in unplaced contigs. Majority of these missense variants (5 variants) was observed in *KRT8* gene coding for Keratin8. These missense variants were distributed in 18 genes namely *BOLA*, *EI24*, *FABP2*, *FOXN3*, *HIST3H2A*, *JSP*.*1*, *KLK4*, *KNG1*, *KRT8*, *LOC616948*, *MDH1B*, *PERM1*, *PPP1R15A*, *SAP18*, *SLC25A36*, *STON2*, *TTC16*, *YME1L1* (Full names of genes are mentioned in Table S2). Further, 7 of these variants from Tissue were found to be present in predicted domains in their respective proteins. Also, most of these variants were predicted to have neutral effect as per PROVEAN except for 5 variants from tissue present in *HIST3H2A*, *FABP2*, *KRT8* and *BOLA* genes. Amongst all the genes with missense variants, *BOLA* and *JSP.1* were known to be involved in antigen presentation and processing; while, *KRT8*, *EI24*, *PPP1R15A* and *SAP18* were known to be involved in apoptosis.

**Table 1:** Details of the non-synonymous variants found in the study. No domain = no domain present at site of mutation; No predicted domain = no domains predicted by ScanProsite in the protein

Chromosome	Position	Reference	Alternate	Sample observed in	Variant details	Gene	dbSNP ID/remarks	Domain at variant position as per ScanProsite	Profiles with high probability of occurrence	PROVEAN score, Prediction (cutoff = −2.5)
chr7	2,464,501	G	A	Blood	c.343G > A p.Val115Met	*HIST3H2A*	rs110683125	No domain		−1.566, Neutral
chr10	95,036,026	A	G	Blood	c.803 T > C p.Leu268Ser	*STON2*	novel	No domain	N-glycosylation site	0.156, Neutral
chr16	48,710,798	G	A	Blood	c.1216G > A p.Ala406Thr	*PERM1*	rs132949042; different variant in different assembly	No predicted domain		−0.322, Neutral
chr18	56,842,100	G	A	Blood	c.46C > T p.Leu16Phe	*KLK4*	rs207879743	No domain		−1.194, Neutral
chr1	82,070,747	C	G	Tissue	c.935C > G p.Ala312Gly	*KNG1*	rs43705174	Kininogen-type cystatin domain; Cysteine proteases inhibitors signature		−1.941, Neutral
chr1	130,202,956	C	T	Tissue	c.596G > A p.Ser199Asn	*SLC25A36*	novel	Solute carrier (Solcar) repeat	Protein kinase C phosphorylation site	−1.306, Neutral
chr2	99,269,960	C	G	Tissue	c.355G > C p.Glu119Gln	*MDH1B*	rs209212339	No predicted domain		−1.616, Neutral
chr6	6,791,420	G	C	Tissue	c.173G > C p.Ser58Thr	*FABP2*	rs43450379; no missense variant details available	No domain		0.71, Neutral
chr6	6,791,467	G	C	Tissue	c.220G > C p.Ala74Pro	*FABP2*	rs209700321, rs43450380; no missense variant details available	No domain	Casein kinase II phosphorylation site	−3.07, **Deleterious**
chr7	2,464,214	C	G	Tissue	c.56C > G p.Ser19Cys	*HIST3H2A*	novel	No domain	Protein kinase C phosphorylation site	−3.878, **Deleterious**
chr10	75,128,451	T	A	Tissue	c.1112A > T p.Lys371Met	*KRT8*	novel	Intermediate filament (IF) rod domain		−5.471, **Deleterious**
chr10	75,128,460	C	T	Tissue	c.1103G > A p.Arg368Lys	*KRT8*	novel	Intermediate filament (IF) rod domain		−0.198, Neutral
chr10	75,128,467	C	T	Tissue	c.1096G > A p.Ala366Thr	*KRT8*	novel	Intermediate filament (IF) rod domain		−3.519, **Deleterious**
chr10	75,128,854	C	T	Tissue	c.709G > A p.Glu237Lys	*KRT8*	novel	Intermediate filament (IF) rod domain		−1.535, Neutral
chr10	75,129,398	C	T	Tissue	c.165G > A p.Met55Ile	*KRT8*	novel	No domain		−0.094, Neutral
chr10	103,727,085	C	T	Tissue	c.158G > A p.Gly53Glu	*FOXN3*	rs208438463	No domain		−2.22, Neutral
chr11	101,809,760	C	G	Tissue	c.2298C > G p.Asp766Glu	*TTC16*	novel	No domain	Casein kinase II phosphorylation site	0.715, Neutral
chr12	35,754,313	T	G	Tissue	c.493A > C p.Thr165Pro	*SAP18*	novel	No predicted domain		1.027, Neutral
chr13	16,992,516	T	G	Tissue	c.83A > C p.Asn28Thr	*YME1L1*	rs209048457	No domain		−0.964, Neutral
chr15	47,201,978	G	C	Tissue	c.380C > G p.Ala127Gly	*LOC616948*	rs209504468	Zinc finger B-box type		4.546, Neutral
chr18	55,254,392	C	A	Tissue	c.1057C > A p.Pro353Thr	*PPP1R15A*	novel	No predicted domain		1.818, Neutral
chr23	27,580,458	T	G	Tissue	c.433A > C p.Ile145Leu	*JSP.1*	rs42311131	No domain		−1.142, Neutral
chr23	27,580,953	A	T	Tissue	c.134 T > A p.Ile45Asn	*JSP.1*	novel	No domain		0.088, Neutral
chr23	27,637,398	T	C	Tissue	c.535A > G p.Arg179Gly	*BOLA*	another variant of same position exists (rs135180180, leading to ter codon)	No domain		−2.647, **Deleterious**
chr29	30,399,904	A	G	Tissue	c.1012A > G p.Thr338Ala	*EI24*	rs42178224	No predicted domain	Protein kinase C phosphorylation site	0.308, Neutral

## Discussion

Two of the genes carrying missense variant, ***BOLA*** and ***JSP.1***, are involved in antigen binding and are part of MHC class-I molecules. Protein from these genes control immune response and as per KEGG pathways, are involved in various pathogen related response pathways including viral myocarditis, antigen processing and presentation, epstein-barr virus infection, phagosomes and viral carcinogenesis. Multiple mutations in both these genes could hinder the antigen presentation and promote external factor (like pathogens) based tumour. Although, mutation in *BOLA* gene (p.R179G) was predicted to be deleterious by PROVEAN server, mutations detected in both *BOLA* (p.R179G) and *JSP.1* (p.I145L, p.I45N) gene were not present within any predicted domains as per ScanProsite.

Another missense variant containing gene ***EI24*** (Etoposide-induced protein 2.4) also known as p53-induced gene 8 protein (*PIG8*) is one of the tumour-suppressing gene induced by p53 during apoptosis [25]. In humans, the gene is located on chromosome 11 q23-q25, a region associated with the frequent alterations in cancers [26, 27]. Previous study has found this gene to contain a large proportion of mutations in human breast cancer cells and predicted this gene to be a mutational target in human cancers [28]. In our study the observed mutation is part of Protein kinase C phosphorylation site on the protein. ***PPP1R15A*** codes for protein phosphatase 1 regulatory subunit 15A also known as growth arrest and DNA damage-inducible protein (*GADD34*). The protein is involved in suppression of cell growth and in ER stress-induced cell death in humans [29, 30]. ***SAP18*** (Sin3A associated protein 18) is yet another protein involved in apoptosis. *SAP18* forms a tetrameric complex with *RNPS1* and Acinus termed as *ASAP* (apoptosis- and splicing-associated protein) complex. *ASAP* complex was predicted to participate in both apoptosis and RNA splicing [31].

Furthermore, Keratin is an important constituent of horns and hoofs of animals. Keratin is an intermediate filament, a type of cytoskeletal fibrillary proteins, having diameter of 8 to 11 nm. There are around twenty Keratins classified under two broad types (Type-I and Type-II) of Keratins and are commonly expressed in type-I/type-II pairs [32]. The most common pair *K8*/*K18* is expressed in almost all epithelial cells. Also, *K8*/*K18* are an essential part of apoptotic cycle and linked with Fas-induced apoptosis [33, 34]. Various studies in mouse hepatocytes have revealed that mutations or knockdown of *K8* have resulted in diseases including tumorigenesis [34–37]. We observed 5 missense variants in ***KRT8*** gene producing *K8* protein. Further, 4 of these variants (p.K371M, p.R368K, p.A366T, p.E237K) were located within Intermediate filament (IF) rod domain of KRT8 protein and 2 (p.K371M, p.A366T) were predicted to have deleterious effect.

We included samples from blood to compensate for the unavailability of well-annotated genome of *Bos indicus*. Since, we used genome of *Bos taurus* as a reference, there is a chance that some of the variants could be variation among two species of cattle. However, those variants should be observed across all the samples irrespective of sample source. We observed that missense variants in our study were present either in tissue or blood samples only. Furthermore, variants in the genes playing part in immunity or apoptosis, as discussed above, were observed in tissue samples which is the exact point of tumour.

## Conclusion

We demonstrated an efficient approach for SNP discovery in targeted exonic approach. Bioinformatic variant analysis resulted in total 30 and 96 cancer specific SNPs out of which 4 and 21 missense variants were found in blood and tissue, respectively. *KRT8* was found to be apex gene having five missense variants. Involvement of *KRT8* gene in horn constituent and apoptotic cycle directs its role in horn cancer tumorigenesis. Similarly, mutation in immune response related genes namely *BOLA* and *JSP.1* suggest their possible role in bypassing the immune response to cancer. These genes’ association with event of Horn cancer reflect their potential to be considered for genetic marker. The present finding would provide base for further screening of genes and identification of marker for early diagnosis and therapeutics intervention of horn cancer.

## Supplementary Information

**Additional file 1: Table S1** Statistics of data generated and processed. **Table S2**: List of genes and their abbreviations.

## Data Availability

The datasets generated and/or analysed during the current study are available in the Sequence Read Archive database in NCBI and can be accessed through accession numbers SRX4946233-SRX4946292.

## Abbreviations

SCC: Squamous Cell Carcinoma
SNP: Single Nucleotide Polymorphism
NGS: Next Generation Sequencing
