# Supplementary material for: Molecular portrait of squamous cell carcinoma of the bovine horn evaluated by high-throughput targeted exome sequencing: a preliminary report
Source: BMC Vet Res. 2020 Nov 26;16:461. doi: 10.1186/s12917-020-02683-y (PMC7690171; doi:10.1186/s12917-020-02683-y)
Supplement: Supplementary file 1 — Additional file 1: Table S1 Statistics of data generated and processed. Table S2: List of genes and their abbreviations. [file 12917_2020_2683_MOESM1_ESM.pdf]

Supplementary files

Supplementary tables:

Table S1: Statistics of data generated and processed

		Blood				Tissue			
		Total reads generated	Mapped paired-reads	On-target mapping (paired-reads)	Variants predicted	Total reads generated	Mapped paired-reads	On-target mapping (paired-reads)	Variants predicted
Cancer	HC-01	3,594,487	3,547,397 (98.69%)	1,615,249(44.94%)	27,893	5,932,669	5,901,286 (99.47%)	3,149,360(53.09%)	65,199
	HC-02	3,761,874	3,704,983 (98.49%)	1,484,310(39.46%)	24,327	4,446,692	4,422,622 (99.46%)	2,220,391(49.93%)	39,345
	HC-03	5,517,249	5,466,719 (99.08%)	2,384,614(43.22%)	41,818	4,787,012	4,761,073 (99.46%)	2,422,418(50.6%)	47,462
	HC-04	6,006,032	5,947,823 (99.03%)	2,692,117(44.82%)	48,920	4,837,859	4,813,768 (99.5%)	2,472,538(51.11%)	48,390
	HC-05	4,949,089	4,909,191 (99.19%)	2,116,224(42.76%)	38,336	4,430,531	4,404,665 (99.42%)	2,244,470(50.66%)	39,315
	HC-06	5,618,967	5,556,698 (98.89%)	2,301,580(40.96%)	39,368	4,966,944	4,941,157 (99.48%)	2,291,614(46.14%)	42,920
	HC-07	4,017,976	3,963,574 (98.65%)	1,501,298(37.36%)	22,564	4,250,605	4,225,824 (99.42%)	2,146,093(50.49%)	38,247
	HC-08	4,546,856	4,484,634 (98.63%)	1,764,427(38.81%)	30,293	3,776,606	3,763,430 (99.65%)	1,889,648(50.04%)	30,540
	HC-11	5,268,407	5,195,699 (98.62%)	2,308,758(43.82%)	44,543	3,611,084	3,581,797 (99.19%)	1,696,739(46.99%)	22,167

	HC-12	5,069,916	4,981,221 (98.25%)	2,184,138(43.08%)	42,588	4,637,473	4,603,984 (99.28%)	2,191,159(47.25%)	38,333
	HC-13	4,915,711	4,861,340 (98.89%)	1,514,136(30.8%)	22,532	5,207,413	5,170,605 (99.29%)	2,339,930(44.93%)	43,012
	HC-14	4,639,647	4,578,971 (98.69%)	1,648,410(35.53%)	25,986	3,511,748	3,484,220 (99.22%)	1,651,577(47.03%)	20,046
	HC-15	5,128,793	5,066,155 (98.78%)	2,165,126(42.22%)	40,641	4,780,963	4,746,771 (99.28%)	1,978,868(41.39%)	38,182
	HC-16	3,828,372	3,814,869 (99.65%)	1,651,855(43.15%)	27,775	4,767,738	4,719,665 (98.99%)	1,999,456(41.94%)	38,392
	HC-17	8,233,203	8,182,620 (99.39%)	1,651,924(20.06%)	25,202	4,020,617	4,003,202 (99.57%)	1,582,770(39.37%)	28,509
	HC-18	9,016,577	8,918,745 (98.91%)	3,492,591(38.74%)	58,531	5,292,243	5,273,145 (99.64%)	2,728,383(51.55%)	55,343
	HC-19	7,145,994	7,079,351 (99.07%)	2,553,019(35.73%)	40,434	5,687,072	5,662,272 (99.56%)	2,732,332(48.04%)	52,944
	HC-22	6,143,693	6,064,049 (98.7%)	2,066,454(33.64%)	32,654	5,748,939	5,723,969 (99.57%)	3,120,224(54.27%)	61,562
	HC-23	5,326,214	5,271,729 (98.98%)	2,483,848(46.63%)	51,405	5,414,680	5,391,103 (99.56%)	2,905,331(53.66%)	57,850
	HC-25	3,399,781	3,337,468 (98.17%)	1,424,972(41.91%)	15,136	6,060,037	6,037,857 (99.63%)	3,301,342(54.48%)	67,073
	HC-26	4,021,088	3,974,323 (98.84%)	1,392,835(34.64%)	18,962	5,409,200	5,386,083 (99.57%)	2,914,001(53.87%)	55,594
	HC-27	4,378,659	4,317,760 (98.61%)	2,111,478(48.22%)	42,578	4,974,396	4,944,593 (99.4%)	2,657,488(53.42%)	47,884

	HC-28	3,752,506	3,732,243 (99.46%)	1,760,430(46.91%)	28,437	6,647,523	6,617,419 (99.55%)	3,161,402(47.56%)	64,113
	HC-29	6,702,460	6,608,206 (98.59%)	3,507,516(52.33%)	69,071	6,560,236	6,526,143 (99.48%)	3,244,085(49.45%)	64,783
	HC-30	4,135,594	4,118,341 (99.58%)	1,973,502(47.72%)	34,656	4,472,620	4,451,999 (99.54%)	2,142,935(47.91%)	38,924
Normal	HN-01	4,145,504	4,123,654 (99.47%)	2,023,423(48.81%)	35,452	4,055,268	4,036,097 (99.53%)	1,958,346(48.29%)	33,293
	HN-02	4,977,963	4,953,435 (99.51%)	2,408,318(48.38%)	51,456	2,718,997	2,705,063 (99.49%)	1,291,247(47.49%)	13,106
	HN-03	5,273,440	5,249,033 (99.54%)	2,111,117(40.03%)	43,342	4,255,381	4,236,542 (99.56%)	2,053,436(48.26%)	35,767
	HN-04	5,355,691	5,330,078 (99.52%)	2,508,033(46.83%)	52,551	3,359,428	3,343,790 (99.53%)	1,617,018(48.13%)	20,722
	HN-05	4,417,744	4,394,979 (99.48%)	2,042,240(46.23%)	42,645	3,526,941	3,501,700 (99.28%)	1,691,390(47.96%)	24,186

Table S2: List of genes and their abbreviations

Gene	Gene Name
BOLA	MHC class I antigen
EI24	EI24, autophagy associated transmembrane protein
FABP2	fatty acid binding protein 2, intestinal
FOXN3	forkhead box N3
HIST3H2A	histone cluster 3, H2a
JSP.1	MHC class I antigen
KLK4	kallikrein related peptidase 4
KNG1	kininogen 1
KRT8	keratin 8
LOC616948	tripartite motif-containing protein 5-like
MDH1B	malate dehydrogenase 1B
PERM1	*PPARGC1* and *ESRR* induced regulator, muscle 1
PPP1R15A	protein phosphatase 1 regulatory subunit 15A
SAP18	Sin3A associated protein 18
SLC25A36	solute carrier family 25 member 36
STON2	stonin 2
TTC16	tetratricopeptide repeat domain 16
YME1L1	YME1 like 1 ATPase
